# 达沙替尼导致大量蛋白尿一例

**DOI:** 10.3760/cma.j.issn.0253-2727.2021.08.017

**Published:** 2021-08

**Authors:** 国辉 李, 聪 刘, 炜炜 秦, 柰岑 周, 怀鹏 郭, 学倩 严, 月茹 及, 怡 陈, 利 刘, 英民 梁

**Affiliations:** 1 西安国际医学中心医院血液病医院，西安 710100 Hospital of Hematology, Xi'an International Medical Center, Xi'an 710100, China; 2 空军军医大学唐都医院血液科，西安 710032 Tangdu Hospital, Air Force Military Medical University, Xi'an 710038, China

患者，女，28岁。2008年8月因“纳差、乏力、腹胀1周”就诊于当地医院。血常规：WBC 75.7×10^9^/L，PLT 413×10^9^/L，HGB 119 g/L，遂转入我院。查体：脾大，肋缘下5 cm；染色体核型：46，XX，t（9;22）（q34；q11）[10]；BCR-ABL融合基因（P210）阳性；骨髓细胞形态学及活检均支持慢性髓性白血病（CML）。诊断为CML（慢性期，Sokal评分：低危），给予伊马替尼400 mg/d。3个月后达血液学完全缓解，随后未监测染色体及BCR-ABL融合基因。

2014年7月自觉胸闷、气促，血常规：WBC 12.23×10^9^/L，PLT 3020×10^9^/L，HGB 115 g/L。再次入住我科，腹部超声示脾大，脾厚5.9 cm，骨髓细胞形态学及活检提示CML加速期，BCR-ABL/ABL为98.6％，染色体核型：46，XX，t（9;22）（q34；q11）[3]/46，idem，+ der（22）t（9;22）（q34;q11）[3]。ABL激酶区未见突变，诊断为CML加速期，给予达沙替尼100 mg/d联合高三尖杉酯碱及阿糖胞苷化疗，血液学完全缓解后出院，院外口服达沙替尼100 mg/d。2016年3月外周血BCR-ABL融合基因未测出（ABL拷贝数262 000），随后BCR-ABL融合基因持续为0。

患者2017年8月出现眼睑水肿，晨轻暮重，休息后无明显缓解，尿蛋白（+++），诊断为“慢性肾炎”，予“黄葵胶囊”等口服。2017年9月反复发生胸腔积液，停用达沙替尼，并每月复查BCR-ABL融合基因，期间复查尿蛋白（+）。5个月后复查BCR-ABL融合基因0.058％，遂再次服用达沙替尼，随后未规律检测尿蛋白。

2019年9月患者再次出现颜面水肿，尿常规示蛋白（++++）。24 h尿蛋白定量3540 mg，肝肾功能均正常，总胆固醇3.59 mmol/L（正常参考值3.10～5.17 mmol/L），甘油三酯2.31 mmol/L（正常参考值<1.7 mmol/L），高密度脂蛋白0.69 mmol/L（正常参考值>0.91 mmol/L），低密度脂蛋白1.85 mmol/L（正常参考值2.7～3.1 mmol/L）。血常规：WBC 3.91×10^9^/L，RBC 4.2×10^12^/L，HGB 118 g/L，PLT 196×10^9^/L。骨髓细胞形态学未见异常，BCR-ABL融合基因为0。肾活检：可见5个肾小球，未见肾小球球性硬化，其中1个肾小球阶段性硬化，其余肾小球系膜细胞和基质轻度弥漫性增生，系膜区无明显嗜复红蛋白沉积。免疫荧光：未见免疫复合物沉积。电镜：基底膜弥漫性偏薄，厚度大约250 nm，阶段性内皮下间隙增宽，足突大部分融合，未见电子致密物沉积，形态考虑薄基底膜肾病。患者停用达沙替尼，更换为伊马替尼400 mg/d口服。1年后尿蛋白（+），24 h尿蛋白350 mg。截至2020年12月，患者BCR-ABL融合基因持续为0。

讨论：达沙替尼导致大量蛋白尿临床罕见，目前国外报道10例，本例是我国达沙替尼导致大量蛋白尿首例报道。达沙替尼导致的蛋白尿多见于女性，与原发病无关。蛋白尿最快于达沙替尼治疗后2周出现，最慢为58个月。患者均表现为肾病性蛋白尿，而血肌酐及肌酐清除率均在正常范围，临床表现更接近肾病综合征。达沙替尼停药后，尿蛋白一般1个月内消失，最快3 d。患者肾脏病理改变均累及足细胞足突，表现为不同程度足细胞足突的消失、融合，肾小球基底膜改变主要表现为局灶性皱褶、变薄，内皮损伤较为少见，表现为内皮细胞肿胀。其中，足细胞的损伤可能为其最主要的作用。分子机制可能是达沙替尼抑制了LIMK激酶活性，从而影响了PAK-LIMK信号轴和肌动蛋白的调节，导致cofillin磷酸化降低和肌动蛋白纤维形成。

